# A New Nomogram Prediction Model for Left Ventricular Thrombus in Patients with Left Ventricular Aneurysm after Acute Myocardial Infarction

**DOI:** 10.31083/j.rcm2510377

**Published:** 2024-10-23

**Authors:** Yuanzhen Xu, Zhongfan Zhang, Daoyuan Si, Qian Zhang, Wenqi Zhang

**Affiliations:** ^1^Department of Cardiology, China-Japan Union Hospital of Jilin University, Jilin Provincial Molecular Biology Research Center for Precision Medicine of Major Cardiovascular Disease, 130033 Changchun, Jilin, China

**Keywords:** acute myocardial infarction, left ventricular aneurysm, left ventricular thrombus, nomogram prediction model

## Abstract

**Background:**

To identify the factors influencing the development of a left ventricular thrombus (LVT) in patients with a left ventricular aneurysm (LVA) after acute myocardial infarction (AMI) and to utilize these variables to establish a new nomogram prediction model for individual assessment in LVT.

**Methods:**

We screened data on 1268 cases of LVA at the China-Japan Union Hospital of Jilin University between January 1, 2018 and December 31, 2023, and identified a total of 163 LVAs after AMI. The independent risk factors of LVT in patients with LVA after AMI were identified from univariable and multivariable logistic regression analyses and a nomogram prediction model of LVT was established with independent risk factors as predictors. We used the area under the curve (AUC) and a calibration curve to determine the predictive accuracy and discriminability of nomograms. Furthermore, decision curve analysis (DCA) was utilized to further validate the clinical effectiveness of the nomogram.

**Results:**

Multivariate logistic regression analysis identified that preoperative thrombus in myocardial infarction 0, left ventricular diameter, and anterior wall myocardial infarction were independent risk factors of LVT in patients with LVA after AMI (*p* < 0.05). The nomogram prediction model constructed using these variables demonstrates exceptional performance, as evidenced by well-calibrated plots, favorable results from DCA, and the AUC of receiver operating characteristic (ROC) analysis was 0.792 (95% CI: 0.710–0.874, *p* < 0.01).

**Conclusions:**

A new nomogram prediction model was developed to enable precise estimation of the probability of LVT in patients with LVA after AMI, thereby facilitating personalized clinical decision-making for future practice.

## 1. Introduction 

The development of a left ventricular aneurysm (LVA) is a common complication following acute myocardial infarction (AMI), characterized by the protrusion of the left ventricular wall, which consists of mature scar tissue. The prevalence of LVA in patients with coronary artery disease was found to be 7.6%, whereas the incidence of LVA in patients with AMI was significantly higher at 28.0% [1, 2]. The development of a left ventricular thrombus (LVT) typically occurs following the onset of an LVA, which can result in severe systemic embolism, disability, or even fatality, without any apparent warning signs [3]. Although numerous studies have demonstrated that LVA is a prominent risk factor for LVT formation [4, 5], there is currently no definitive evidence to suggest that LVA inevitably leads to LVT.

The nomogram models are based on multivariate analysis and extensively integrate the results of logistic or Cox regression to predict the probability of a specific clinical event in patients, accompanied by intuitive graphical representations. A growing body of literature has highlighted the advantages of these models in predicting mortality and other prognostic outcomes [6, 7]. Compared to conventional evaluation methods, the nomogram model can provide more accurate and intuitive predictions. However, there is currently no existing literature reporting individualized prediction models for assessing LVT in patients with LVA.

In this study, we conducted an analysis of the risk factors associated with LVT after AMI in patients with LVA. Additionally, we developed a nomogram model based on these identified risk factors to predict the occurrence of LVT in patients with LVA.

## 2. Methods

### 2.1 Study Population

**Fig. 1.**
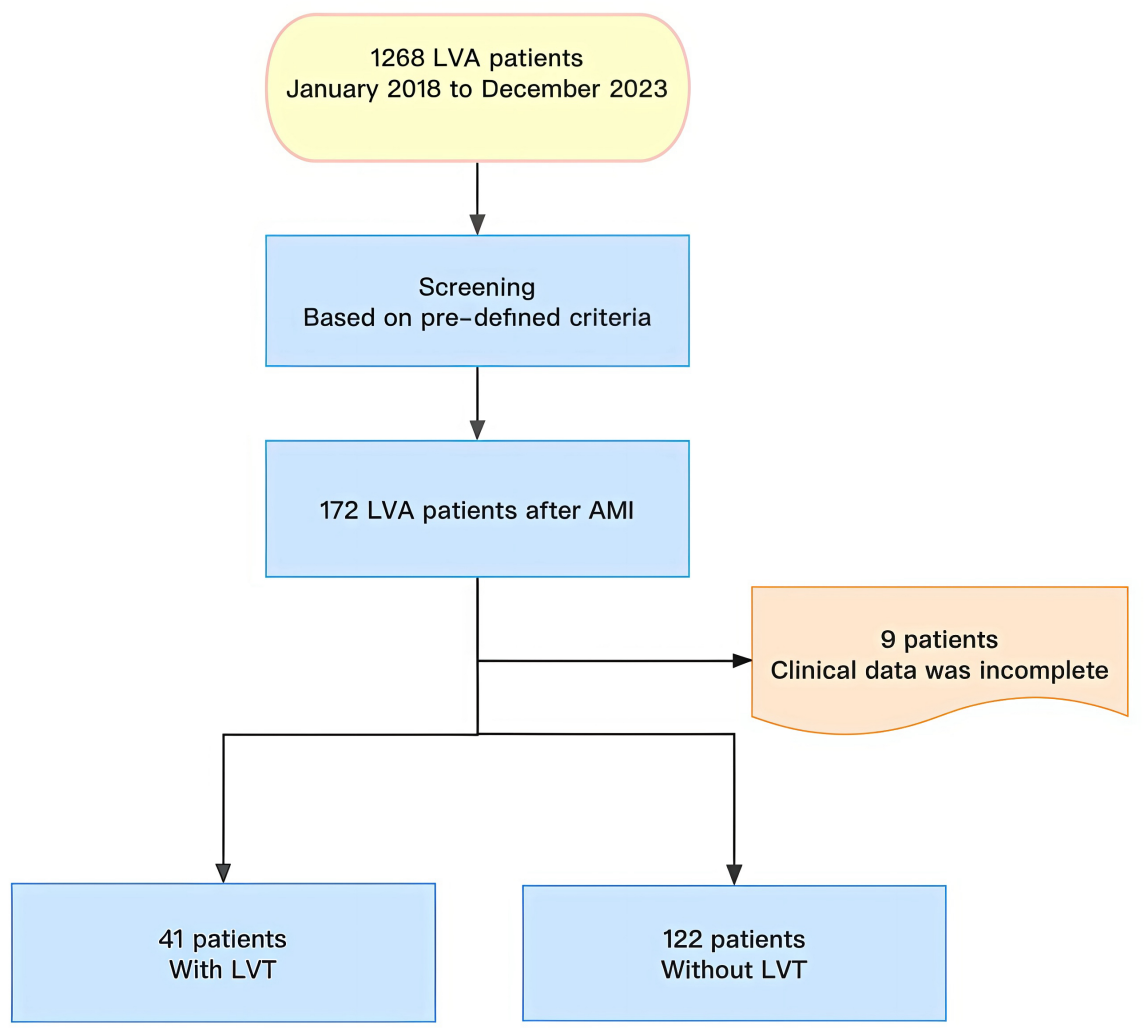
**Study chart flow**. LVA, left ventricular aneurysm; AMI, acute myocardial infarction; LVT, left ventricular thrombus.

In this retrospective study conducted at a single center, all the enrolled patients were individuals with LVA after AMI, who were admitted to China-Japan Union Hospital of Jilin University between January 2018 and December 2023. This study flow chart is depicted in Fig. 1.

The diagnosis of AMI is based on the criteria outlined in the fourth edition of the general definition of myocardial infarction [8]. Diagnostic criteria for LVA in transthoracic echocardiography (TTE) include: (1) Thinning of the ventricular wall leading to outward expansion, accompanied by cystic or irregular pathological changes; (2) Presence of a well-defined hypoechoic area with clear demarcation connecting to both the cardiac cavity and surrounding myocardium; (3) Visualization of disturbed and turbulent blood flow within the cardiac cavity [9]. LVT observed by TTE was defined as a distinct mass (1) that echoes in the left ventricular cavity that was seen clearly throughout the cardiac cycle with a structural texture different from the myocardium; (2) which is contiguous with the endocardium in an area of abnormal wall motion; (3) which can be separated from the underlying endocardium by an endocardial lining [10].

Exclusion criteria: (1) patients with incomplete TTE examination that precluded grouping; (2) patients with severe impairment of major organ function, such as liver, kidney, and lung; (3) patients with malignant tumors expected to have a survival time of less than 3 months; (4) patients with immune system disorders and infectious diseases; (5) pregnant patients; (6) patients with psychiatric abnormalities; (7) non-consenting participants.

The study only included patients who had confirmed LVA and LVT by two independent cardiologists and had documented routine follow-up. In cases of disagreement among the cardiologists conducting the review of LVA and LVT images, an additional independent cardiologist was consulted to validate the final assessment. The requirement for written consent was waived due to the minimal risk posed to patients and the retrospective nature of the study design. This study was approved by the Ethics Committee of China-Japan Union Hospital of Jilin University.

### 2.2 Baseline and Data Collection

The factors previously associated with prognosis after myocardial infarction, including age, gender, height, weight, body mass index (BMI), presence of hypertension and diabetes, smoking and drinking history, history of myocardial infarction, stroke or other embolisms, previous percutaneous coronary intervention (PCI) or heart failure; Killip classification and myocardial infarction classification; anticoagulation measures during hospitalization; use of an angiotensin-converting enzyme inhibitor (ACEI), angiotensin II receptor blocker (ARB), angiotensin receptor neprilysin inhibitor (ARNI), or β-blocker; PCI duration, number of coronary artery lesions and culprit vessels involved as well as degree of stenosis in the culprit vessel; whether thrombus aspiration was conducted during the procedure; thrombolysis in myocardial infarction (TIMI) flow grade before revascularization. Additionally measured parameters included left atrium diameter (LAD), interventricular septum (IVS), left ventricular diameter (LVD), left ventricular posterior wall thickness (LVPW), right ventricular diameter (RVD), pulmonary artery systolic pressure (PA), left ventricular ejection fraction (LVEF); areas for mitral regurgitation area, tricuspid regurgitation area, aortic regurgitation area, and pulmonary regurgitation area. Laboratory parameters consisted of triglycerides (TG), total cholesterol (TC), low density lipoprotein (LDL), high density lipoprotein (HDL), troponin I (TnI), N-terminal pro brain natriuretic peptide (NT-proBNP), D-Dimer, fibrinogen (FIB), hemoglobin (HB), mean platelet volume (MPV) and serum creatinine (SCR). The baseline characteristics were obtained from the hospital’s electronic medical record system.

### 2.3 Statistical Analysis 

Baseline characteristics were presented as either continuous or categorical variables. The categorical variables were statistically described using frequency and percentage, while the continuous variables were described using mean ± standard deviation (for normally distributed data) or median (P25, P75) (for skewed distribution), respectively. Categorical variable data were expressed as n (%) and compared between groups using either the χ^2^ test or Fisher’s exact probability method. All clinical covariates were evaluated through univariate logistic regression analysis to determine their significant association with outcomes. Covariates with a *p*-value of ≤0.05 in the univariate models were included in the multivariate logistic regression models to identify independent risk factors for outcomes and these independent risk factors were utilized as predictors for constructing a nomogram. The accuracy of the nomogram was evaluated using receiver operating characteristic (ROC) analysis, discriminative power was verified through calibration plots, and decision curve analysis (DCA) was employed to demonstrate the relationship between false positive and true positive scores at different risk thresholds. Statistical significance was considered at *p* < 0.05. SPSS 27.0 (IBM SPSS statistics, Chicago, IL, USA) and R software (version 4.3.2, R Foundation for Statistical Computing, Vienna, Austria) were used for statistical analysis in this research.

## 3. Results

A total of 41 (25.15%) patients with LVA after AMI were identified to develop LVT. The baseline characteristics of patients with diagnosed LVA after AMI are shown in Tables 1,2. The baseline analysis revealed that patients with LVT exhibited a higher prevalence of ST-segment elevation myocardial infarction (STEMI), Killip II, anterior wall myocardial infarction (MI), and a history of smoking compared to the non-LVT group (*p* < 0.05). In terms of TTE, patients with LVT exhibited a lower LVEF compared to those without LVT and tended to have larger LVD (*p* < 0.05).

**Table 1.** **Baseline characteristics in patients with two groups**.

Characteristics	LVT (n = 41)	Non-LVT (n = 122)	*p*‐value
Age, n (%)	66.00 (57.00–73.00)	68.00 (60.00–74.00)	0.243
Male, n (%)	30 (73.17)	69 (56.56)	0.059
BMI, kg/m^2^	23.64 ± 3.49	24.30 ± 3.88	0.332
Hypertension, n (%)	19 (46.34)	64 (52.46)	0.498
Diabetes, n (%)	10 (24.39)	53 (43.44)	0.030
History of MI, n (%)	0 (0.00)	3 (2.46)	0.573
History of stroke, n (%)	4 (9.76)	28 (22.95)	0.066
Other embolisms, n (%)	0 (0.00)	4 (3.28)	0.573
History of PCI, n (%)	11 (26.83)	12 (9.84)	0.007
History of HF, n (%)	5 (12.20)	16 (13.11)	0.879
Smoke, n (%)	9 (21.95)	2 (1.64)	0.001
Drink, n (%)	22 (53.66)	65 (53.28)	0.966
Hyperlipidemia, n (%)	26 (63.41)	62 (50.82)	0.162
Atrial fibrillation, n (%)	0 (0.00)	3 (2.46)	0.573
Newly developed HF, n (%)	4 (9.76)	28 (22.95)	0.066
STEMI, n (%)	38 (92.68)	90 (73.77)	0.011
Killip I, n (%)	13 (31.71)	54 (44.26)	0.157
Killip II, n (%)	21 (51.22)	41 (33.61)	0.044
Killip III, n (%)	5 (12.20)	20 (16.39)	0.519
Killip IV, n (%)	2 (4.90)	7 (5.74)	1.000
Anterior wall MI, n (%)	35 (85.37)	71 (58.20)	0.002
Inferior wall MI, n (%)	3 (7.32)	19 (15.57)	0.181
High lateral wall MI, n (%)	0 (0.00)	1 (0.82)	1.000

LVT, left ventricular thrombus; BMI, body mass index; MI, myocardial infarction; PCI, percutaneous coronary intervention; HF, heart failure; STEMI, ST-segment elevation myocardial infarction.

**Table 2.** **Clinical characteristics in patients with two groups**.

Characteristics	LVT (n = 41)	Non-LVT (n = 122)	*p*-value
LVEF, %	42.20 (33.00–50.10)	45.00 (38.40–54.00)	0.137
LAD, mm	40.80 (37.70–45.50)	39.10 (34.80–42.60)	0.059
RVD, mm	21.80 (20.30–24.00)	22.00 (20.0–23.15)	0.143
IVS, mm	10.40 (8.90–12.30)	10.00 (9.00–11.90)	0.537
LVD, mm	54.70 (46.20–58.60)	49.40 (44.70–55.00)	0.033
LVPW, mm	10.40 (8.50–11.60)	9.70 (9.00–11.00)	0.669
PA, mm	22.70 (21.00–24.60)	22.60 (20.89–24.40)	0.233
Mitral RA, cm^2^	2.00 (0.00–4.20)	2.00 (0.00–4.90)	0.530
Tricuspid RA, cm^2^	0.00 (0.00–2.80)	0.00 (0.00–2.65)	0.891
Aortic RA, cm^2^	0.00 (0.00–0.00)	0.00 (0.00–0.75)	0.288
Pulmonary RA, cm^2^	0.00 (0.00–0.00)	0.00 (0.00–0.00)	0.378
TG, mmol/L	1.67 (1.04–2.18)	1.54 (1.09–2.14)	0.977
TC, mmol/L	4.43 (3.15–5.80)	4.61 (3.74–6.02)	0.159
LDL, mmol/L	2.73 (1.98–3.58)	2.95 (2.24–3.84)	0.419
HDL, mmol/L	0.96 (0.81–1.20)	1.08 (0.89–1.30)	0.152
SCR, µmol/L	88.10 (78.30–112.50)	81.70 (65.85–104.60)	0.490
eGFR, µmol/L	71.50 (58.10–85.76)	78.00 (54.85–90.80)	0.573
TnI, ng	0.61 (0.06–5.20)	1.36 (0.15–11.85)	0.124
D-Dimer, mg/L	0.86 (0.53–1.30)	0.60 (0.30–1.53)	0.182
NT-proBNP, pg/mL	2330.00 (1090.00–5180.00)	3000.00 (565.50–6793.23)	0.728
FIB, mg/dL	3.35 (2.80–4.53)	3.40 (2.79–4.15)	0.931
HB, g/L	142.00 (128.00–158.00)	138.00 (125.50–150.00)	0.481
MPV, fL	9.90 (9.40–11.10)	9.70 (9.15–10.60)	0.324
PCI, n (%)	35 (85.37)	105 (86.07)	0.911
Anticoagulation, n (%)	35 (85.37)	96 (78.69)	0.352
Anticoagulation time, days	5.00 (3.00–7.00)	5.00 (3.00–6.75)	0.931
ACEI, n (%)	10 (24.39)	28 (22.95)	0.850
ARB, n (%)	2 (4.88)	6 (4.92)	1.000
ARNI, n (%)	13 (31.71)	29 (23.77)	0.315
β-blockers, n (%)	32 (78.04)	90 (73.77)	0.585

LVT, left ventricular thrombus; LVEF, left ventricular ejection fraction; LAD, left atrium diameter; RVD, right ventricular diameter; IVS, interventricular septum; LVD, left ventricular diameter; LVPW, left ventricular posterior wall thickness; PA, pulmonary artery systolic pressure; RA, regurgitation area; TG, triglycerides; TC, total cholesterol; LDL, low density lipoprotein; HDL, high density lipoprotein; SCR, serum creatinine; TnI, troponin I; FIB, fibrinogen; HB, hemoglobin; MPV, mean platelet volume; eGFR, estimated glomerular filtration rate; PCI, percutaneous coronary intervention; ACEI, angiotensin converting enzyme inhibitor; ARB, angiotensin II receptor blocker; ARNI, angiotensin receptor neprilysin inhibitor; NT-proBNP, N-terminal pro brain natriuretic peptide.

**Fig. 2.**
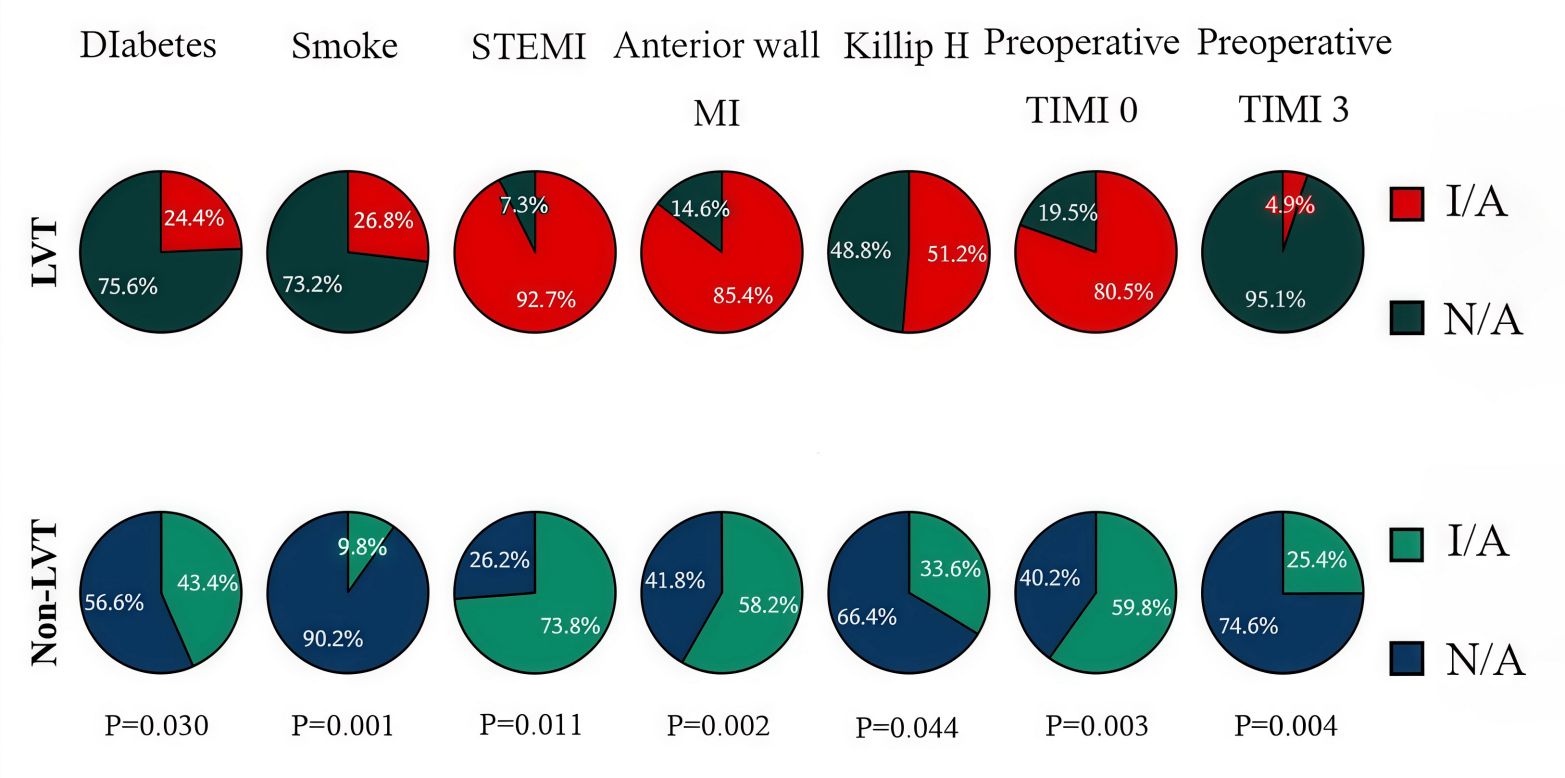
**Categorical pie chart**. LVT, left ventricular thrombus; STEMI, ST-segment elevation myocardial infarction; MI, myocardial infarction; TIMI, thrombolysis in myocardial infarction; I/A, is applicable; N/A, not applicable.

**Fig. 3.**
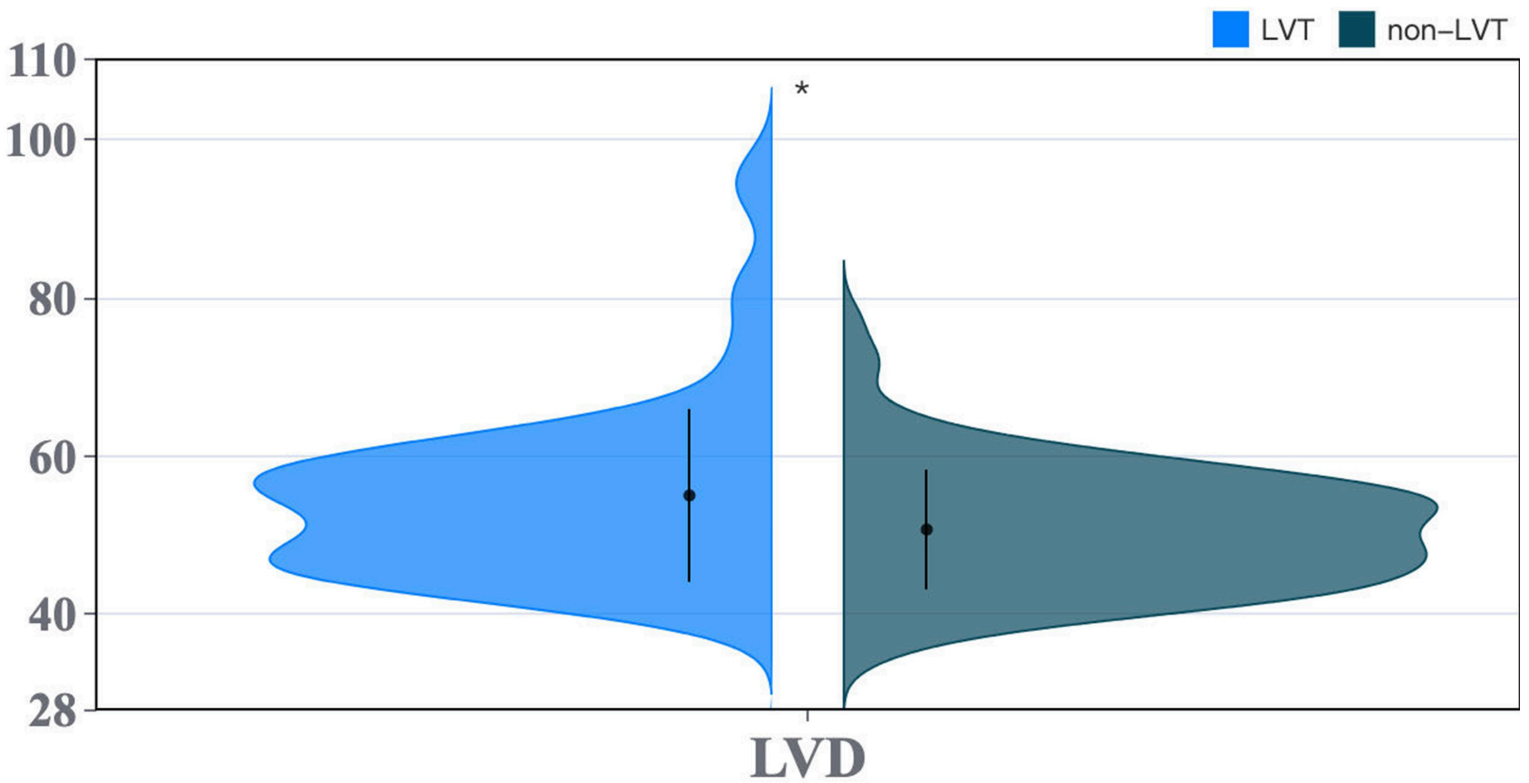
**Violin plot**. LVT, left ventricular thrombus; LVD, left ventricular diameter.

The data regarding treatment during hospitalization and each intra operative coronary angiography of the two groups were presented in Table 3. A total of 140 (85.89%) patients received PCI. There were 35 patients (85.37%) in the LVT group and 122 patients (86.07%) in the non-LVT group. The degree of vascular stenosis was significantly higher in the LVT group, and there was also a higher proportion of patients with preoperative TIMI grade of 0 (TIMI 0) (*p* < 0.05). To visually represent the variables exhibiting statistically significant differences, we employed categorical pie chart and violin plot (Figs. 2,3).

**Table 3.** **Intra operative coronary angiography in patients with two groups**.

Characteristics	LVT (n = 35)	Non-LVT (n = 105)	*p-*value
PCI duration, min	40.00 (34.00–66.00)	50.00 (35.00–82.00)	0.195
LM, n (%)	3 (8.57)	7 (6.67)	0.711
LAD, n (%)	31 (88.57)	83 (79.05)	0.243
LCX, n (%)	19 (54.29)	69 (65.71)	0.226
RCA, n (%)	21 (60.00)	67 (63.81)	0.686
Multivessel disease, n (%)	30 (75.00)	87 (82.86)	0.693
Coronary artery narrow degree, n (%)	100.00 (100.00–100.00)	100.00 (99.00–100.00)	0.005
Preoperative TIMI 0, n (%)	33 (94.29)	73 (69.52)	0.003
Preoperative TIMI 1, n (%)	0 (0.00)	0 (0.00)	1.000
Preoperative TIMI 2, n (%)	0 (0.00)	1 (0.95)	1.000
Preoperative TIMI 3, n (%)	2 (5.71)	31 (29.52)	0.004
Thrombus aspiration, n (%)	1 (2.86)	5 (4.76)	1.000

LM, left main artery; LAD, left anterior descending artery; LCX, left circumflex artery; RCA, right coronary artery; TIMI, thrombolysis in myocardial infarction; LVT, left ventricular thrombus; PCI, percutaneous coronary intervention.

**Fig. 4.**
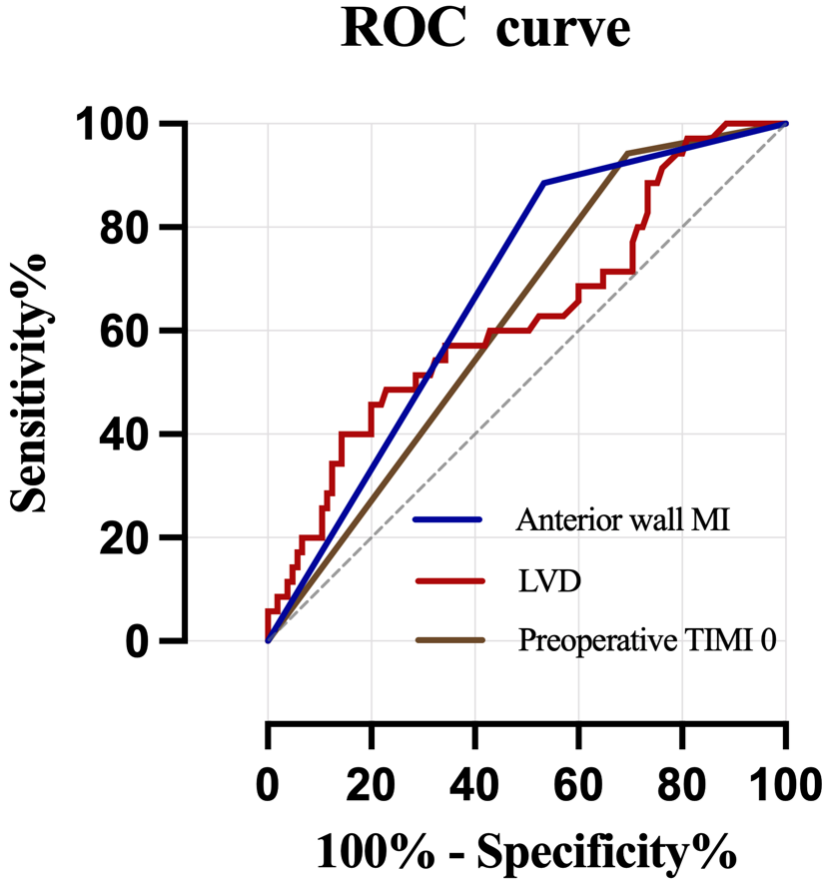
**ROC curve of three variables on LVT prediction**. ROC, receiver operating characteristic; LVT, left ventricular thrombus; TIMI, thrombolysis in myocardial infarction; MI, myocardial infarction; LVD, left ventricular diameter.

**Fig. 5.**
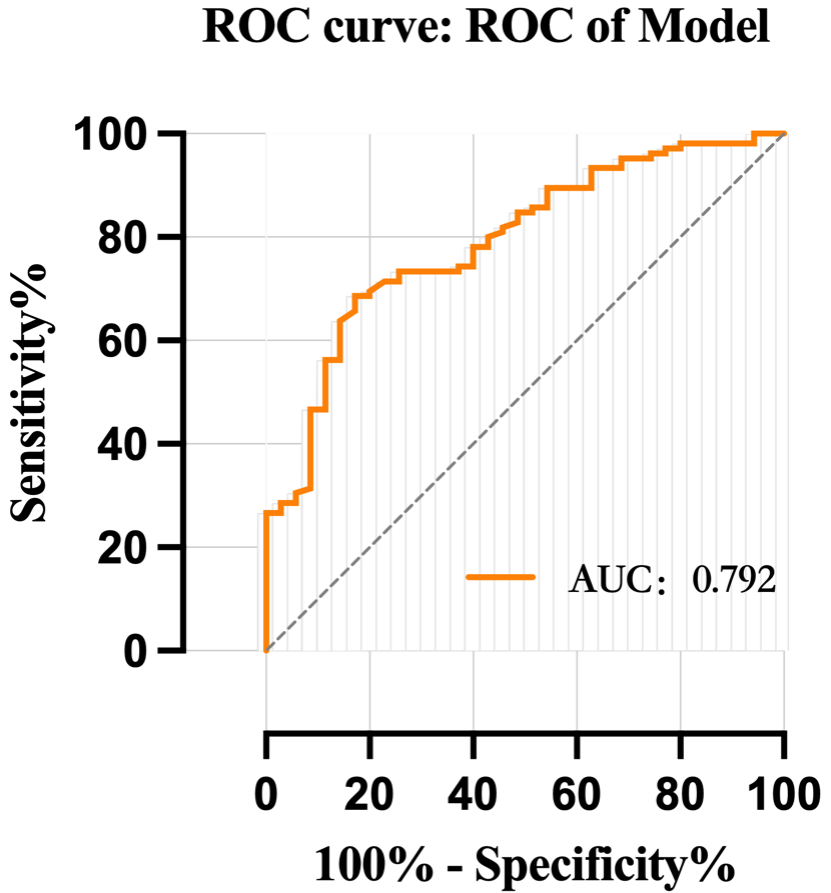
**ROC curve of model**. ROC, receiver operating characteristic; AUC, area under curve.

In this study, five variables including smoking history, preoperative TIMI 0, Killip II, LVD, and anterior wall MI were selected from a total of variables in patients with LVA after AMI. Subsequent logistic regression analysis demonstrated that preoperative TIMI 0, LVD, and anterior wall MI contribute as independent risk factors (OR values: 7.778, 1.053 and 6.095; *p* < 0.05) (Table 4). Based on these three independent risk factors, a multifactorial logistic regression analysis was employed to develop a predictive model for the occurrence of LVT. The ROC curves of independent risk factors and the prediction model were plotted in Fig. 4 and Fig. 5. The prediction model exhibited an AUC of 0.792 (95% CI: 0.710–0.874, *p* < 0.01), as shown in Table 5.

**Table 4.** **Logistic regression analysis of LVT in patients with LVA after AMI**.

Variables	β	S.E.	Wald x^2^	*p-*value	OR	95% CI
Anterior wall MI	1.808	0.612	8.734	0.003	6.095	1.838–20.211
Preoperative TIMI 0	2.051	0.809	6.428	0.011	7.778	1.593–37.977
Killip II	0.384	0.456	0.710	0.399	1.469	0.601–3.590
Smoke	0.796	0.528	2.271	0.132	2.217	0.787–6.241
LVD	0.052	0.025	4.289	0.038	1.053	1.003–1.105

LVT, left ventricular thrombus; LVA, left ventricular aneurysm; AMI, acute myocardial infarction; MI, myocardial infarction; TIMI, thrombosis in myocardial infarction; LVD, left ventricular diameter.

**Table 5.** **Predictive Value of independent risk factors**.

Variables	Cut-off	Sensitivity	Specificity	YI	AUC	95% CI	*p-*value
Model	0.300	0.829	0.686	0.515	0.792	0.710–0.874	<0.001
LVD	55.450	0.463	0.779	0.242	0.630	0.520–0.740	0.022
Anterior wall MI	0.500	0.886	0.467	0.353	0.676	0.582–0.771	0.002
Preoperative TIMI 0	0.500	0.943	0.305	0.248	0.624	0.526–0.722	0.029

LVD, left ventricular diameter; MI, myocardial infarction; TIMI, thrombosis in myocardial infarction; YI, Youden’s index; AUC, area under curve.

**Fig. 6.**
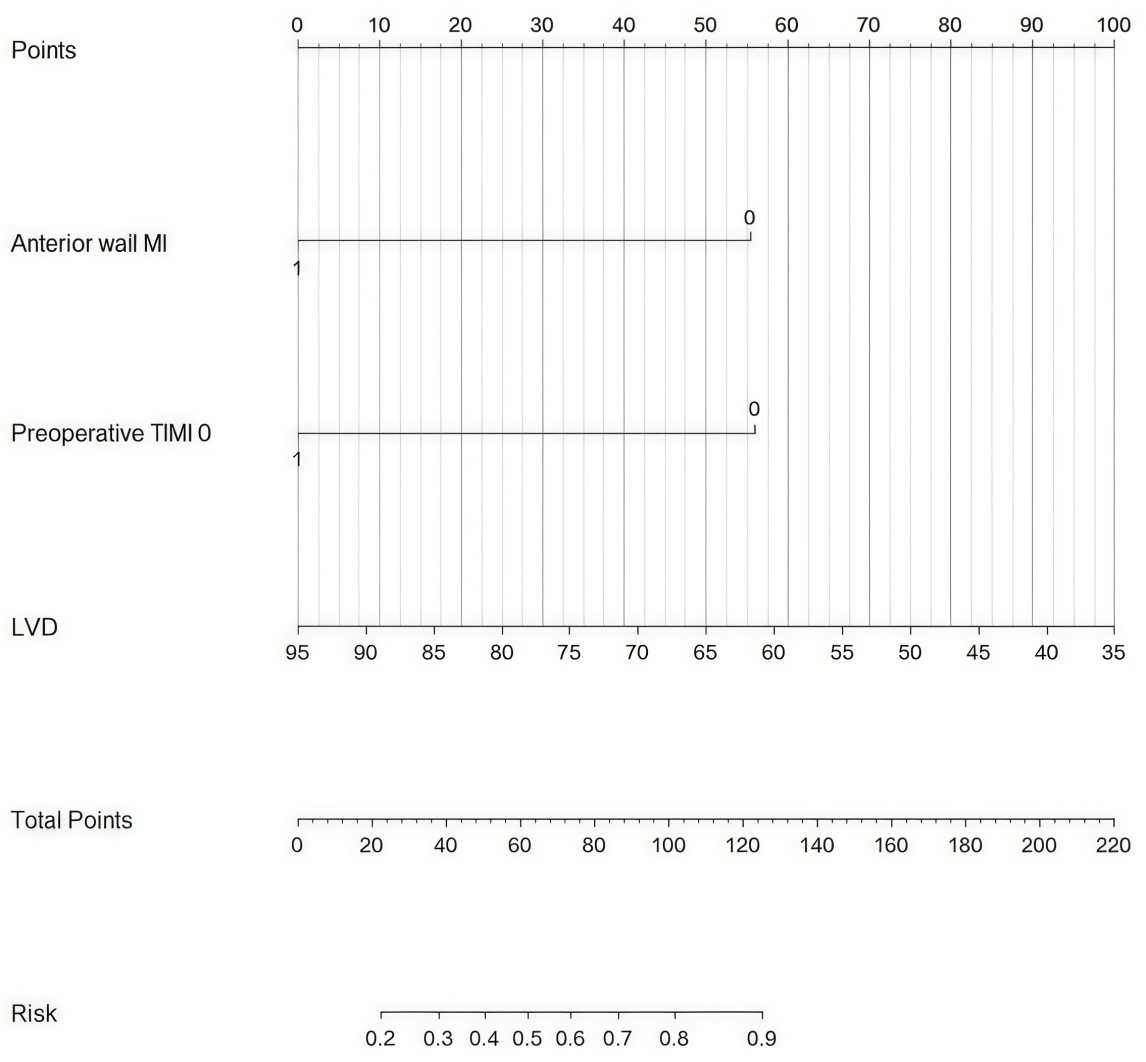
**Nomogram**. MI, myocardial infarction; TIMI, thrombolysis in myocardial infarction; LVD, left ventricular diameter.

**Fig. 7.**
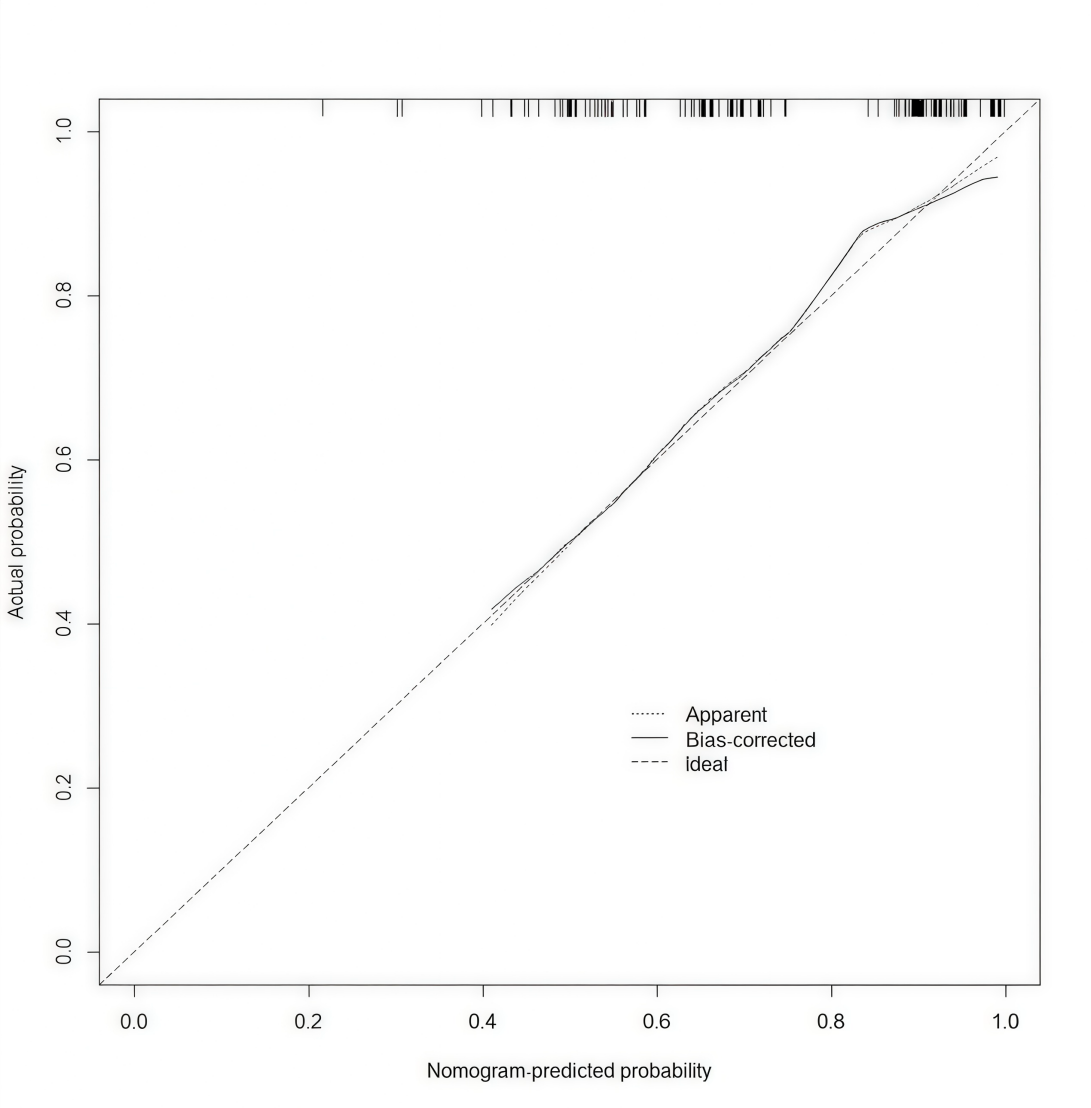
**Calibration curve**.

**Fig. 8.**
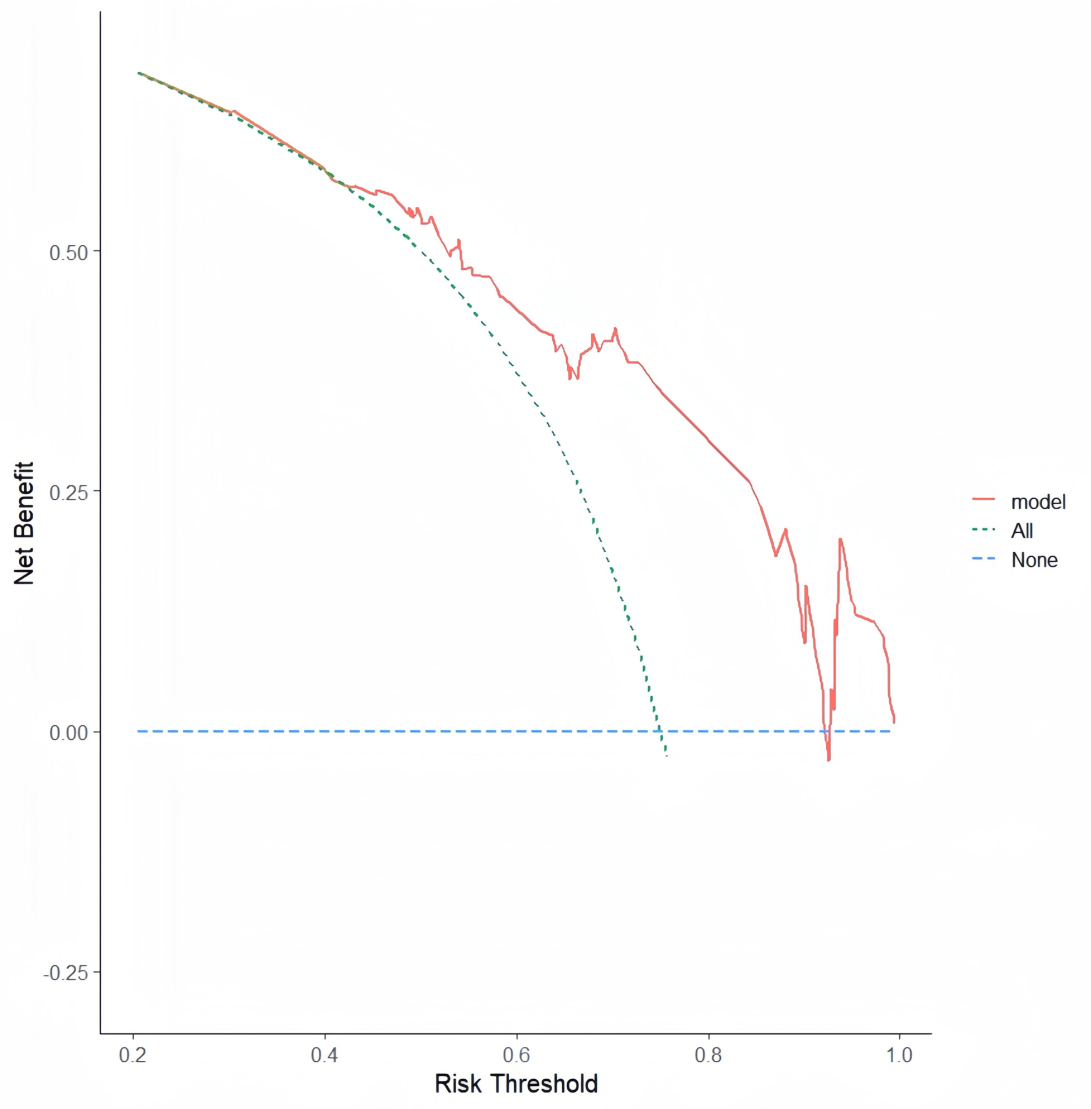
**Decision curve**.

In this study, a nomogram for LVT (Fig. 6) was constructed based on the prediction model which can effectively enhance preoperative assessment capabilities in patients with LVT by providing a visually intuitive representation of prediction outcomes. The calibration curve of the nomogram demonstrated a robust concordance between observed data and predicted values (Fig. 7). The decision curve illustrated in Fig. 8 demonstrates a positive net benefit in predicting LVT, thereby highlighting its exceptional clinical utility.

## 4. Discussion

In recent years, advancements in reperfusion therapy, antiplatelet, and anticoagulant therapy have led to a significant reduction in the incidence of complications following AMI. However, there was no corresponding decrease in mortality from complications following AMI. LVA is a common complication following AMI, and LVT typically occurs after the formation of LVA. Due to the lack of reported data on this specific subset of patients, we conducted a retrospective study. To the best of our knowledge, our research represents the first retrospective study to establish a nomogram model for predicting LVT in patients with LVA after AMI. Furthermore, the ROC curve, calibration plot, and DCA collectively demonstrate the robust predictive capacity of the nomogram. It is anticipated that the utilization of this model in clinical practice will assist physicians in determining the most suitable treatment approach for their patients.

We screened data from 1268 cases of LVA and identified 163 cases which occurred within one month of AMI. The incidence of LVT in these cases was found to be 25.15%, which is consistent with previous findings [11, 12]. A prospective study revealed that during a span of 3 months, 22% of patients with anterior wall MI also developed LVA [13]. LVA is also associated with high cardiogenic mortality, with rates of about 67% at 3 months and 80% at 1 year [14]. LVT is another complication that may arise after AMI. In an analysis involving over 10,000 STEMI patients, it was discovered that the overall incidence of LVT was approximately 2.7%. However, the incidence of LVT in patients with anterior wall MI was notably higher at around 9.1% [15]. It was reported that the one-year all-cause mortality rate for patients with LVT is 13%, and the incidence of embolic events is approximately 1.9% [16]. LVT typically occurs following LVA. It is hypothesized that local turbulence, caused by sluggish blood flow in the LVA, may initiate the development of LVT. This can further result in the thinning of the infarction area and expansion of damaged endothelial cells, ultimately leading to an increase in the volume of LVA. This process forms a vicious cycle. Any single complication can significantly impact the prognosis of patients with AMI, particularly as they often occur concurrently. Therefore, it is crucial to promptly identify the contributing factors that may lead to LVT and to implement effective preventive measures for patients with LVA after AMI. It is imperative to recognize these factors in order to ensure the well-being of patients and to minimize the risk of further complications.

In this study, multivariate logistic regression analysis revealed that preoperative TIMI 0, anterior wall MI, and LVD were independent risk factors for predicting LVT in patients with LVA after AMI. Anterior wall MI has been consistently identified as a significant risk factor for LVT in numerous studies, and our research further substantiates this conclusion [17]. The myocardium’s anterior wall plays a significant role in the pumping function, with its primary source of blood supply being the left anterior descending artery. In cases where the artery is a single vessel and lacks collateral circulation, there is an increased likelihood of widespread infarction occurring. This can result in dyskinesia under pressure within the heart cavity, potentially leading to localized blood clot formation. Due to inadequate blood supply to the coronary arteries, the ventricular wall in this area gradually thinned and expanded, leading to the development of LVA and eventually incorporating LVT. Preoperative TIMI 0 was identified as an additional independent risk factor in this study. It is hypothesized that the cause may be the complete occlusion of blood vessels in individuals with preoperative infarction, leading to a continuous state of ischemia, hypoxia, and metabolic disorders of cardiomyocytes. This ultimately leads to an increased size of myocardial infarction and diastolic dysfunction, ultimately resulting in LVT. The ROC curve of this study revealed that LVD >55.45 had a significant predictive value for the occurrence of LVT in patients with LVA after AMI. Previous research has established that left ventricular systolic dysfunction is a strong predictor of LVT after AMI [18, 19]. Therefore, it can be inferred that the exacerbation of LVD further worsens the blood stasis in the left ventricular apex. As the left and right ventricles share the ventricular septal wall, dysfunction in the left ventricle may further decrease the contractile capacity of the upper segment of the interventricular septum, leading to impaired hemodynamics in pulmonary circulation and reduced filling of the left ventricle. We believe that there is likely a close relationship between ventricular cardiac dysfunction and pulmonary effusion thrombosis in these patients.

Currently, the management of LVT in patients with LVA after AMI is categorized into pharmacological therapy and interventional therapy [20, 21]. No matter which treatment option is chosen, the clinician’s experience and accurate judgment are essential. In this study, we have developed a new comprehensive evaluation system to accurately assess LVT. We utilized commonly observed risk factors to construct a nomogram, which serves as a visual representation of the data and aids in facilitating clinical decision-making. Our study possesses several notable advantages compared to previous experiments. Firstly, the inclusion of a substantial number of cases of LVA in patients with AMI significantly enhances the relevance and applicability of the model. Secondly, the integration of supplementary routine preoperative serological indicators and intraoperative coronary angiography effectively enhances clinicians’ assessment and evaluation. Thirdly, through ROC curve analysis, it is demonstrated that the combined predictors exhibit superior accuracy in predicting the risk of LVT compared to independent influencing factors. Fourthly, our model demonstrates a high degree of goodness-of-fit and predictive value. Finally, given its inherent simplicity, it is highly likely that this approach will be extensively employed in clinical settings.

### Limitations

We retrospectively collected clinical data from patients undergoing LVA treatment after AMI in the Jilin Province. However, it is important to note that there are geographical disparities in the incidence of AMI, which may be attributed to factors such as regional economic status and dietary patterns. In addition, a few limitations of our study need to be addressed. First, currently unidentified potential predictors were not included. Second, thrombus mobility and thrombus protrusion, which are associated with thromboembolism, were not extensively investigated. Third, the findings only pertain to the index stay period and cannot be extrapolated to events which occurred after discharge. Meanwhile, the actual detection rate of LVT and the incidence of thromboembolism or bleeding events may have been underestimated. Finally, due to the retrospective design of this study, selection bias may exist. Therefore, further prospective studies are warranted, and the sample size should be expanded by collaborating with other centers.

## 5. Conclusions

In this study, we developed a new nomogram prediction model to effectively predict the likelihood of LVT in patients with LVA after AMI. The model incorporates common risk factors, including anterior wall MI, preoperative TIMI 0, and LVD. It can aid clinicians in determining the necessity of early intervention based on individual patient conditions and preoperative predictive outcomes.

## Availability of Data and Materials

The datasets used and/or analyzed in this study are available from the corresponding author upon reasonable request.
